# The importance of Continuing Professional Development of clinical competence in municipal healthcare: a cross-sectional study of workplace-based training

**DOI:** 10.1186/s12912-026-04546-7

**Published:** 2026-04-15

**Authors:** Ingrid Taylor, Linn Hege Førsund, Gøril Nonstad Jevne, Monica Samuelsen, Dag Hofoss, Pia Cecilie Bing-Jonsson

**Affiliations:** 1 Department of Nursing and Health Sciences, University of South-Eastern Norway, Borre, Norway; 2 Ringerike Municipality, Intermunicipal Ringerike Region, Health and Care Services, Ringerike, Norway; 3 Department of Welfare, Hole Municipality, Hole, Norway

**Keywords:** Clinical competence, Competence measurement, Continuing professional development, Workplace-based training, Municipal healthcare, Registered nurses, Assistant nurses

## Abstract

**Background:**

Healthcare personnel face increasing demands on their clinical competence due to shifting responsibilities, growing task complexity, and ongoing policy changes. In response, structured workplace-based training programmes have become a central component of lifelong learning and Continuing Professional Development. This study was conducted at the start of the Clinical Observation Competence in the Municipal Health Service programme, a national initiative in Norway designed to strengthen clinical observation and decision-making skills among healthcare personnel.

**Methods:**

A cross-sectional design was used, involving 811 healthcare personnel from six municipalities. Participants included registered nurses, assistant nurses, and assistants. Clinical competence was assessed using the validated Ms. Olsen test, which measures knowledge across three domains: Acute Help, Nursing Measures, and Involving Physician. Statistical analyses included ANOVA and multiple linear regression to examine group differences and predictors.

**Results:**

Registered nurses scored significantly higher than assistant nurses and assistants across all domains, with the strongest performance in Acute Help. Assistant nurses outperformed assistants in Acute Help and Involving Physician, but no significant difference was found in Nursing Measures. All groups showed lower scores in Nursing Measures, indicating a critical gap. Professional role was the strongest predictor of competence, while position size had a more modest effect. Work experience was positively associated with competence in the domain Acute Help, while age was not a significant factor. Healthcare personnel working in home care services scored higher than those in nursing homes.

**Conclusion:**

The study reveals a clear professional hierarchy in clinical competence and highlights specific gaps, particularly in Nursing Measures. The unexpected finding that home care personnel outperformed those in nursing homes may suggests a shift in competence patterns. These results underscore the importance of structured, workplace-based training programmes to address competence gaps and support professional development in municipal healthcare.

**Supplementary Information:**

The online version contains supplementary material available at 10.1186/s12912-026-04546-7.

## Introduction

Lifelong learning is essential in today’s knowledge-based society, involving continuous, voluntary, and self-directed acquisition of knowledge and skills across various life contexts through both formal and informal means [1]. In the healthcare sector, continuous competence development plays a crucial role in lifelong learning. It contributes to both patient safety and the consistent delivery of high-quality care [2–5]. Continuing Professional Development (CPD) provides a structured, lifelong learning process that enables healthcare personnel to maintain and improve their competence to meet advancing standards of practice and improving patient outcomes [6]. In municipal healthcare, healthcare personnel face increased demands due to shifting responsibilities, growing task complexity, and ongoing organizational and policy changes that requires continuous adaptation [7]. In response to these evolving demands, CPD is increasingly recognized as a key mechanism for supporting healthcare personnel in maintaining competence and adapting to changing care requirements [3, 4]. To ensure the relevance and effectiveness of such training, it must address the actual competence needs of health personnel [8]. This study examines the baseline clinical competence of municipal healthcare personnel participating in the Program for Clinical Observation in the Municipalities (ClinObsMun), a structured workplace-based CPD initiative in Norway.

## Background

### Workplace-based training

The WHO Global Patient Safety Action Plan 2021–2030 identifies CPD as a strategic priority, emphasizing the importance of structured, workplace-based training for all healthcare personnel [3]. Such training has been shown to be effective and plays a critical role in strengthening both individual capabilities and organizational outcomes [9, 10]. Despite widespread agreement on the value of workplace-based training, a review highlights a lack of consensus on the effectiveness of individual training initiatives and the extent to which learning is applied in practice [11]. This issue is especially relevant in dynamic work environments like healthcare, where the clinical competence of healthcare personnel can deteriorate over time [10, 12, 13].

WHO calls on healthcare institutions to develop and implement CPD initiatives using an interprofessional learning approach [3]. These initiatives should include specialized programs that focus on team-oriented and task-specific approaches, such as bedside instruction and simulation-based learning, with formal recognition upon successful completion [3, 4]. Such training aligns with broader lifelong learning frameworks that emphasize the integrating of learning into daily work life [1, 8, 9]. Thus, formal learning activities must also be continuously reinforced and embedded into daily work practices to achieve a lasting impact [11, 14]. However, such programs require significant investment for municipalities [15], and leaders often face challenges when evaluating their effectiveness and impact on the quality of care [4, 16]. Without systematic evaluation, demonstrating the value of competence development initiatives and tailoring training to actual learning needs becomes challenging [9, 15].

### Learning theory

To understand how learning can be effectively integrated into practice, it is useful to consider key learning theories. Experiential learning theory emphasizes learning through experience, reflection, and application, making hands-on methods such as simulation and case-based training particularly relevant [17]. Sociocultural learning theory builds on Vygotsky’s ideas, emphasizing that learning is fundamentally social and occurs through interactions with more knowledgeable individuals [18]. Together, these learning theories highlight the importance of mentorship, peer collaboration, and guided workplace training, which are central concepts in the Communities of Practice framework [19]. The framework describes how learning evolves through shared experience and problem-solving within professional groups. In healthcare, fostering such collaborative environments is crucial for continuous competence development and high-quality care. Furthermore, transfer of training theory emphasizes that newly acquired knowledge must be reinforced in practice to ensure lasting impact [20]. This is especially important in healthcare, where the ability to apply learning directly impacts patient outcomes. Together, the theories highlight that learning in healthcare depends on context, practice, and social interaction.

### Clinical competence

While learning theories explain how competence can be developed, a clear understanding of what competence means in clinical practice is equally important. As healthcare environments grow more complex, the ability to apply learning effectively in real-life situations becomes vital. Clinical competence encompasses the knowledge, skills, and attitudes essential for professional practice, with evidence-based nursing at its core [5]. The International Council of Nursing [ICN] defines clinical competence as the ability to make correct decisions in critical situations, ensure patient safety, and demonstrate appropriate skills [21]. Nurses, including registered nurses (RNs) and assistant nurses (ANs), must be able to observe, evaluate, and make decisions, based on changes in a patient’s health and condition [2, 13]. Building clinical competence in municipal healthcare improves the professional status of the field, creates clearer career pathways, and ultimately improves patient outcomes [22].

### The Clinical Observation Competence in the Municipal Health Service program

In response to the growing complexity of healthcare needs in municipal healthcare, workplace-based training plays a vital role in ensuring that lifelong learning and CPD translate into improved clinical competence in daily practice. The Clinical Observation Competence in the Municipal Health Service program (ClinObsMun) is one such initiative. It is designed to strengthen clinical observation skills and reinforce decision-making in municipal healthcare [23]. By integrating workplace-based training with practical competence and skill development, it aligns with the principles of lifelong learning and CPD. ClinObsMun focuses on systematic patient observation using the ABCDE (Airway, Breathing, Circulation, Disability, Exposure) methodology [24], risk assessment, ethical considerations, communication skills, ensuring appropriate care during patient changes, deterioration, or acute illness. The primary target audience includes both trained and as untrained healthcare personnel, with adult patients in institutions, housing, and home-based services as the end users. The first step in the ClinObsMun program is a theoretical course recommended for all healthcare personnel, regardless of educational level, across all municipal healthcare services. The course begins with an introduction to the ABCDE principles and vital sign measurements. The focus of this first step is on taking care of elderly patients, individuals with developmental disabilities, and users with substance abuse and mental health challenges. ClinObsMun also addresses inconsistencies in CPR training and recommends the proACT method course [25] and the National Early Warning Score (NEWS 2) [26] for advanced clinical training. The next steps aim to facilitate cross-disciplinary training within municipal healthcare services and across healthcare levels, focusing on decision-making, communication, leadership, and collaboration [23]. ClinObsMun is offered to all Norwegian municipalities through the Centre for Development of Institutional and Home Care Services as part of a national initiative to ensure high-quality nursing and care services throughout Norway [27].

## The study

### Aim

This study examines the competence of healthcare personnel (RNs, Ans, and assistants) in six Norwegian municipalities at the start of the ClinObsMun program. The aim of this study is to explore baseline clinical competence and identify significant differences and predictors of clinical competence among healthcare personnel at the start of the ClinObsMun. The findings may inform the development of targeted content and delivery strategies for workplace-based training initiatives.

## Method

### Design

This cross-sectional study analysis that aims to examine the clinical competence of healthcare personnel at the start of the ClinObsMun program. By collecting data at the initial measurement point, before implementing further training steps, the study provides an overview of the existing clinical competence and readiness of healthcare personnel for handling acute and subacute patient situations. This cross-sectional study was reported in accordance with the STROBE (Strengthening the Reporting of Observational Studies in Epidemiology) checklist for cross-sectional studies (Supplementary information).

### Study setting and sampling

The study was conducted in six municipalities in South-Eastern Norway that are part of an intermunicipal health service collaboration. The study employed a total population sampling strategy, inviting all eligible employees. Approximately 1,900 employees were invited to participate in the training, 811 completed the questionnaire, resulting in a response rate of 42.7%. No information was available regarding reasons for non-participation among those who did not complete the questionnaire. The questionnaire was distributed to all employees because validated scoring keys already existed for RNs and for ANs/assistants. A third key was planned for other professional groups, but this proved infeasible due to substantial heterogeneity in roles. Consequently, 250 respondents, such as physiotherapists and occupational therapists, were excluded because no predefined scoring key was available for their professions, ensuring consistency and comparability across the included groups.

### Instrument

To assess clinical competence, this study used the 19-item ‘Ms. Olsen test’, derived from the 65-item Nursing Older People-Competence Evaluation Tool (NOP-CET) [28, 29]. The Ms. Olsen test measures clinical decision-making competence among nursing staff working in older people’s care [29]. While NOP-CET has been psychometrically evaluated and shown acceptable validity and reliability based on classical test theory, the Ms. Olsen test was first piloted on a small sample and later evaluated on a larger sample using Rasch Measurement Theory, demonstrating good measurement properties [28, 29]. A factor analysis [12], which assessed the construct validity of NOP-CET, identified three competence domains forming the basis of the Ms. Olsen test: Acute Help, Nursing Measures, and Involving Physician (Table 1).

**Table 1:** Corresponding items in the Ms. Olsen test and the NOP-CET sorted under three domains: Acute Help, Nursing Measures and Involving Physician

Acute Help			Nursing Measures			Involving Physician		
Mrs. Olsen item no	NOP-CET item no		Mrs. Olsen item no	NOP-CET item no		Mrs. Olsen item no	NOP-CET item no	
14	17.3	Patient has symptoms of partial paralysis	7	16.7	Patient has reduced appetite and food intake	11	16.11	Patient has much fresh blood in stool
17	17.6	Patient has newly occurred chest pain	6	16.6	Patient’s skin has rash, wounds, is red or itchy	5	16.5	Patient is substantially dehydrated
16	17.5	Patient has changes in sight, hearing, speech, and comprehension	9	16.9	Patient has pain and discomfort in mouth	10	16.10	Patient is incontinent for urine, stings when urinates
13	17.2	Patient has fallen two times previous week	15	17.4	Patient is more tired during the day	8	16.8	Patient is not able to eat
19	17.8	Patient has short attention span and delusions	18	17.7	Patient has lost interest in keeping home in order, sleeps in chair instead of bed	12	17.1	Patient has increased needs to full care over last two days
			2	16.2	Patient coughs, has increased saliva, and respiration frequency above 20/min			
			3	16.3	Patient has irregular pulse increased more than 20/min in last two days			

Note: Items 1 and 4 in the Ms. Olsen test were not included in domains due to their low loading scores

The Ms. Olsen test begins with a brief description of a typical, fictional patient: “Ms. Olsen is 90 years old and generally weakened by age. Imagine that she develops the following symptoms. Please indicate how you would respond when Ms. Olsen, your patient, develops the following symptoms. You may choose one option on each line.”

Following this introduction, the test presents 19 different symptoms that Ms. Olsen might develop. For each item, respondents can choose from the following response categories:

1. No action required.
2. Observe again the following day.
3. Consult a colleague.
4. Nursing intervention immediately.
5. Have the patient assessed by physician.
6. Require acute help in hospital.

When scoring the responses to the described situation, the option “No action required” (1) is never correct and is scored as 0. Depending on the nature of Ms. Olsen’s symptom, one or two of the other response options could be the best option and are scored as 1. Two versions of scoring are used: one for RNs and one for ANs and assistants, as their scopes of practice differ. The score sheet containing the correct answers for each item for RNs and ANs/assistants is provided in Appendix 1.

### Data collection

Data were collected from October 2021 to June 2022 using a secure online questionnaire distributed to healthcare personnel in six collaborating municipalities as part of the ClinObsMun programme. To encourage participation, the project team actively promoted the survey through professional meetings, training sessions, and local events. In addition to responses to the Ms. Olsen test, five demographic variables were collected: age, professional group, workplace (home care, nursing home, or other services), position size, and years of work experience in the health sector.

### Data analysis

A one-way ANOVA was conducted to compare the mean scores of RNs, ANs, and assistants on total competence and the three subscales: Acute Help, Nursing Measures and Involving Physician. Bonferroni post hoc correction (for equal variances) was applied to adjust for multiple comparisons [30]. The level of statistical significance was set at *p* < 0.05. To investigate the factors influencing clinical competence, a multiple linear regression analysis was conducted. The analysis included three subscales as dependent variables: Acute Help, Nursing Measures, and Involving Physician. Additionally, a composite variable, “total competence,” was calculated as the sum of the three subscales and included as an additional dependent variable. The independent variables included five demographic factors: age, professional group (RN, AN, assistant), workplace (home care, nursing home, or other services), position size, and work experience in the health sector. Age and position size were treated as continuous variables, with position size ranging from 0 to 100%. Work experience was categorised as ≤ 5 years and > 5 years to reflect early-career versus experienced personnel. Before performing the regression analysis, the data were assessed for key assumptions, including linearity, normality of residuals, homoscedasticity, and multicollinearity. Linearity was evaluated through scatterplots of residuals, normality was checked using residual histograms and Q-Q plots and variance inflation factors (VIFs) were calculated to assess multicollinearity [30]. No severe violations of the assumptions were detected. Cases with missing data on outcome or predictor variables were excluded from the analyses (listwise deletion).

### Ethical considerations

The study adhered to ethical research guidelines in accordance with the Declaration of Helsinki [31]. Participants were thoroughly informed about the study’s purpose and procedures, and their responses were collected anonymously. Consent was implied through the completion of the questionnaire. The project was well-anchored in the healthcare administration of the participating municipalities ensuring a strong foundation and oversight of the study. Norwegian Agency for Shared Services in Education and Research pre-approved the project on October 21, 2021 (reference number 263924) (serving as compulsory data protection service).

## Results

The analysed responses (*N* = 561) included 23% RNs, 59%, ANs, and 18% assistants. The average age of the respondents was 44 years (ranging from 18 to 69). Regarding workplace, the respondents were mainly employed in nursing homes (39%), followed by home care (32%) and other workplaces, including mental health and substance abuse services and services for people with disabilities (29%). Respondents reported substantial work experience, with an average of 16.3 years (range: 0–48) (see Table 2). Additionally, 79% held positions exceeding 60% of a full-time employment.

**Table 2:** Characteristics of participants

Categorical variable		Count		Percent
Work experience	More than 5 years	472		86.3
	5 years or less	75		13.7
Sum		547		
	Missing	14		
Professional groups	RN	128		22.8
	AN	333		59.4
	Assistant	100		17.8
Sum		561		100
	Missing: 0			
Workplace	Home Care	178		31.8
	Nursing home	218		39.0
	Other services ^a^	163		29.2
Sum		559		100
	Missing: 2			
Continuous variables	Mean years (min-max)	SD	n=	Missing
Age	44 (18-69)	12.39	541	20
Position size	78 (0-100)	27.01	542	19

^**a**^ Other services include mental health and substance abuse services, and services for people with disabilities

### One-way ANOVA Ms. Olsen-scores by professional group

A one-way ANOVA was conducted to compare the performance of registered nurses (RNs), assistant nurses (ANs), and assistants across total competence and three competence areas: Acute Help, Nursing Measures, and Involving Physician (Table 3). As Levene’s test indicated that the assumption of equal variances was met, multiple comparisons were adjusted by Bonferroni’s correction.

### Total clinical competence

For total competence (scale 0–19), the analysis showed that Ms. Olsen scores were significantly related to professional group (F = 46.285 (df 2,558), *p* < 0.001). Judged by its etasquared, the effect size of professional group was 0.142, which, according to Cohen’s criterion [32] counts as a strong effect. This strong effect size indicates that professional group accounts for a substantial proportion of the variance in total competence. RNs (mean: 12.3; *p* < 0.001) significantly outperformed both ANs (mean: 10.1; *p* < 0.001) and assistants (mean: 8.8; *p* < 0.001). ANs performed significantly better than assistants (*p* = 0.001), highlighting a hierarchy in performance, with RNs achieving the highest competence scores for total competence.

### Acute help

For the Acute Help domain (scale 0–5), (as detailed in Table 1), the analysis showed that Ms. Olsen scores were significantly related to professional group (F = 29.354 (df 2,558), *p* < 0.001). Judged by its etasquared, the effect size of professional group was 0.095, which, according to Cohen’s criterion [32] counts as a medium effect. This medium effect size indicates that professional group accounts for a moderate proportion of the variance in Acute Help scores. RNs (mean: 4.4) significantly outperformed both ANs (mean: 3.6 *p* < 0.001) and assistants (mean: 3.1; *p* < 0.001). ANs performed significantly better than assistants (*p* = 0.0431), highlighting a hierarchy in performance, with RNs showing the highest competence in this area.

### Nursing measures

For the Nursing Measures domain (scale 0–7) (as detailed in Table 1), the analysis showed that Ms. Olsen scores were significantly related to professional group (F = 17.895 (df 2,558), *p* < 0.001). Judged by its etasquared, the effect size of professional group was 0.060, which counts as a medium effect Cohen’s criterion [32]. This medium effect size indicates that professional group accounts for a moderate proportion of the variance in Nursing Measures scores.

RNs (mean: 2.5) significantly outperformed both (mean: 1.8; *p* < 0.001) and assistants (mean: 1.5; *p* < 0.001). No significant difference was observed between ANs and assistants (*p* = 0.136).

### Involving physician

For the Involving Physician domain (scale 0–5) (as detailed in Table 1), the analysis showed that Ms. Olsen scores were significantly related to professional group (F = 11.285 (df 2,558), *p* < 0.001). Judged by its etasquared, the effect size of professional group was 0.039, which counts as a small effect Cohen’s criterion [32]. This small effect size indicates that professional group accounts for a modest proportion of the variance in Involving Physician scores. RNs (mean: 3.9) outperformed both ANs (mean: 3.6; *p* = 0.021) and assistants (mean: 3.1; *p* < 0.001). ANs also showed significantly higher performance than assistants (*p* = 0.007), indicating that this area followed a similar pattern to Acute Help, with RNs performed best, followed by ANs and then assistants.

**Table 3:** ANOVA: All professional groups (RNs, ANs and assistants) and correct answers for subscales

Sample	Total competence			Acute Help			Nursing Measures			Involving Physician		
	Mean correct answers (of 19 items)	SD	95% CI	Mean correct answers (of 5 items)	SD	95% CI	Mean correct answers (of 7 items)	SD	95% CI	Mean correct answers (of 5 items)	SD	95% CI
All groups (*n* = 561)	10.2	3.09	10.09–10.60	3.7	1.32	3.58–3.80	1.9	1.37	1.80–2.02	3.5	1.28	3.44–3.65
RNs (*n* = 128):	**12.3**	2.65	11.83–12.76	**4.4**	0.81	4.24–4.53	**2.5**	1.52	2.233–2.76	**3.9**	1.03	3.69–4.06
ANs (*n* = 333):	10.1	2.91	9.75–10.38	3.6	1.32	3.43–3.72	1.8	1.25	1.67–1.94	3.6	1.30	3.42–3.70
Assistants (*n* = 100):	8.8	2.98	8.18–9.36	3.1	1.48	2.88–3.46	1.5	0.13	1.25–1.77	3.1	1.38	2.80–3.35

Note: Bold text indicates highest value for each domain

## Multiple linear regression: Ms Olsen-score by professional group, controlled for job experience (more or less than 5 years), age, job size (% of full time occupation) and type of workplace

### Total clinical competence

The regression model was statistically significant (F = 13,902 (df 2, 521), *p* < 0.001). The model’s adjusted R² was 0.146, indicating that approximately 15% of the variance in total competence could be explained by the predictors (Table 4).

Controlling for the other predictors, both ANs (B = -2.142; 95% CI: -2.765 – -1.519, *p* < 0.001) and assistants (B = -2.597; 95% CI: -3.506 – -1.689; *p* < 0.001) scored significantly lower than RNs in total clinical competence. Given that the mean score for RNs was approximately 12.3 out of 19 possible correct answers, this corresponds to an average of approximately 2 to 2.6 fewer correct answers for ANs and assistants, respectively. Position size was also a significant predictor (B = 0.012; 95% CI: 0.001–0.022; *p* = 0.029). Accordingly, a 100% position was associated with a total competence score that was, on average, approximately 1.1 points higher than that of a 10% position, on a scale ranging from 0 to 19. Working in a nursing home was associated with lower scores compared to working in home care (B = -0.682; 95% CI: -1.273 – -0.091; *p* = 0.024). This corresponds to an average of approximately 0.7 points lower for working in nursing homes than in home care, on a scale ranging from 0 to 19. Other services (mental health and substance abuse services, and services for people with disabilities), age, and having five or more years of work experience were not significant predictors of total competence.

### Acute help

The regression model was statistically significant (F = 13,500 (df 2, 521), *p* < 0.001). The model’s adjusted R² was 0.142, indicating that approximately 14% of the variance in the domain Acute Help could be explained by the predictors.

Controlling for the other predictors, both ANs (B = -0.776; 95% CI: -1.042 – -0.509; *p* < 0.001) and assistants (B = -0.822; 95% CI: -1.211 – -0.434; *p* < 0.001) scored significantly lower than RNs in the domain Acute Help. Given that the mean score for RNs was approximately 4.4 out of 5 possible correct answers, this corresponds to an average of approximately 0.8 fewer correct answers for both ANs and assistants. Position size was also a significant predictor (B = 0.007; 95% CI: 0.002–0.011; *p* = 0.005). Accordingly, a 100% position was associated with a score for Acute Help that was, an average approximately 0.7 points higher than that of a 10% position, on a scale ranging from 0 to 5. Working in a nursing home was associated with lower scores compared to working in home care (B = -0.508; 95% CI: -0.761 – -0.256; *p* < 0.001). This corresponds to an average of approximately 0.5 points lower for working in nursing homes than in home care, on a scale ranging from 0 to 5. Having five or more years of work experience was a significant predictor (B = 0.385; 95% CI: 0.031–0.739; *p* = 0.033), corresponding to an average score approximately 0.4 points higher than that of those with less experience, on a scale ranging from 0 to 5. Age and working in other services were not statistically significant predictors of Acute Help.

### Nursing measures

The regression model was statistically significant (F = 5,410 (df 2, 521), *p* < 0.001). The model’s adjusted R² was 0.055 indicating that approximately 6% of the variance in total competence could be explained by the predictors (Table 4).

Controlling for the other predictors, both ANs (B = -0.951; 95% CI: -1.375 – -0.527; *p* < 0.001) and assistants (B = -0.665; 95% CI: -0.956 – -0.374; *p* < 0.001) scored significantly lower than RNs in the domain Nursing Measures. Given that the mean score for RNs was 2.5 out of 7 possible correct answers, this corresponds to an average of approximately 1 to 0.7 fewer correct answers for ANs and assistants, respectively. Working in a nursing home was associated with lower scores compared to working in home care (B = -0.311; 95% CI: -0.586 – -0.035; *p* = 0.027) and other services (B = -0.368; 95% CI: -0.663 – -0.072; *p* = 0.015). This corresponds to an average of approximately 0.3 to 0.4 points lower for working in nursing homes and other services, respectively, compared to those working in home care, on a scale ranging from 0 to 7. Position size, age, and work experience were not statistically significant predictors of Nursing Measures competence scores.

### Involving physician

The regression model was statistically significant (F = 3,859 (df 2, 521), *p* < 0.001). The model’s adjusted R² was 0.037, indicating that approximately 4% of the variance in total competence could be explained by the predictors (Table 4).

Controlling for the other predictors, both ANs (B = -0.292; 95% CI: -0.565 – -0.014; *p* = 0.036) and assistants (B = -0.413; 95% CI: -0.811 – -0.014; *p* = 0.042) scored significantly lower than RNs in the domain Involving Physician. Given that the mean score for RNs was 3.9 out of 5 possible correct answers, this corresponds to an average of approximately 0.3 to 0.4 fewer correct answers for ANs and assistants, respectively. Position size was also a statistically significant predictor (B = 0.006; 95% CI: 0.002–0.011; *p* = 0.008). Accordingly, a 100% position was associated with a total competence score that was, on average, approximately 0.5 points higher than that of a 10% position, on a scale ranging from 0 to 5. Age, having five or more years of work experience, and workplace were not statistically significant predictors of Involving Physician competence scores.

**Table 4:** Linear regression: Predictors of competence and subscales

Variable	B (Cl 95)	*p*
**Total competence**		
Work experience^*a*^	0.718 (-0.110–1.546)	0.089
Age	− 0.003 (-0.025–0.018)	0.762
Professional groups: ANs^b^	-2.142 (2.765 – -1.519)	< 0.001*
Professional groups: Assistants^b^	-2.597 (-3.506 – -1.689)	< 0.001*
Position size	0.012 (0.001–0.022)	0.029*
Workplace: Nursing home^c^	-0.682 (-1.273 – -0.091)	0.024*
Workplace: Other^c^	− 0.464 (-1.098–0.169)	0.151
**Acute Help**		
Work experience^a^	0.385 (0.031–0.739)	0.033*
Age	-0.006 (-0.016–0.003)	0.189
Professional groups: AN^b^	-0.776 (-1.042 – -0.509)	< 0.001*
Professional groups: Assistants^b^	-0.822 (-1.211 – -0.434)	< 0.001*
Position size	0.007 (0.002–0.011)	0.005*
Workplace: Nursing home^c^	-0.508 (-0.761 – -0.256)	< 0.001*
Workplace: Other services^c^	-0.156 (0.427–0.115)	0.258
**Nursing Measures**		
Work experience^a^	0.067 (-0.319–0.453)	0.734
Age	− 0.002 (-0.012–0.008)	0.672
Professional groups: AN^a^	-0.951 (-1.375 – -0.527	< 0.001*
Professional groups: Assistants^a^	-0.665 (-0.956 – -0.374)	< 0.001*
Position size	-0.004 (-0.009–0.001)	0.127
Workplace: Nursing home^c^	-0.311 (0.586 – -0.035)	0.027*
Workplace: Other services^c^	-0.368 (-0.663 – -0.72)	0.015*
**Involving Physician**		
Work experience^a^	0.207 (-0.156–0.570)	0.264
Age	0.002 (-0.007–0.012)	0.664
Professional groups: AN^b^	-0.292 (-0.565 – -0.014)	0.036*
Professional groups: Assistants^b^	-0.413 (-0.811 – -0.14)	0.042*
Position size	0.006 (0.002–0.011)	0.008*
Workplace: Nursing home^c^	0.095 (-0.164–0.354)	0.470
Workplace: Other services^c^	0.055 (-0.223–0.333)	0.697

Note: ^a^Reference group is work experience more than 5 years ^b^Reference group is RNs. ^c^Reference group is Home care services

## Discussion

This study investigated baseline differences in clinical competence at the individual, group, and contextual levels. At the individual level, work experience was a significant predictor only for the Acute Help domain, while age was not a significant predictor in any domain and position size showed a small but consistent positive effect across all domains of clinical competence. At the group level, RNs consistently outperformed ANs and assistants across all measured areas of competence. At the contextual level, workplace setting emerged as a significant predictor, with healthcare personnel in home care services demonstrating higher competence scores than those working in nursing homes, particularly in the Acute Help domain. These findings are discussed in term of: Individual Competence and Background, Interdisciplinary Team Competence, and Contextual Competence in Care Settings.

### Individual competence and background

To explore clinical competence at the individual level, this section discusses the findings of work experience, age, and position size. Regression analysis showed individual characteristics as modest or inconsistent links to competence, motivating a focus on how to support individuals through targeted, workplace-based development.

When examining the role of work experience, the regression analysis provides a nuanced understanding. This study found no association between total competence, the domains of Nursing Measures and Involving Physician, and work experience of more than 5 years. However, work experience exceeding 5 years was significantly associated with the domain Acute Help. Age was not associated with total competence or any specific domain. Previous research has shown varied findings. While one study noted a negative impact of age on clinical competence and no association between work experience and competence [12], another found a positive association between registered nurses and competence but a negative association for assistant nurses, with a similar pattern for work experience [33]. A positive association between age and competence assessments in nursing care has been reported, but a negative association between age and self-reported communication skills [34]. Age has been found to be associated with competence among assistant nurses, while work experience is more strongly linked to competence among registered nurses [35]. Thus, the variation in research findings suggests that, while individual factors such as age and work experience may be associated with competence, these relationships could also be influenced by the context of practice and the methods used to measure competence. Position size was a significant predictor of competence in this study, although the correlation was weak, consistent with previous research [34]. In terms of workplace-based training, the lack of significant differences may reflect that competence development is not solely an individual process but is also shaped by organizational culture and the availability of opportunities for situated learning [18]. From the perspective of sociocultural learning theory, structured workplace-based training that incorporates reflection, collaboration, and real-world application is crucial for sustaining competence over time. Further research is needed to explore how life experience, whether through age or accumulated work years, can be meaningfully integrated into CPD strategies in healthcare workplaces.

### Interdisciplinary team competence

To explore clinical competence at the interdisciplinary team level, this section discusses differences across professional roles. ANOVA and regression showed a clear hierarchy between RNs, ANs and assistants, with smaller between‑group differences in Nursing Measures, motivating targeted, workplace‑based CPD and peer‑learning approaches within interdisciplinary teams.

Clinical competence was assessed across three competence domains: Acute Help, Nursing Measures, and Involving Physician, among the three groups of healthcare personnel: RNs, ANs and assistants. The regression analysis confirmed a consistent hierarchy in clinical competence, with RNs outperforming both ANs and assistants across all three domains. A similar pattern emerged in both the regression analysis and ANOVA, with ANs demonstrating significantly higher competence than assistants in Acute Help and Involving Physician, while no significant difference was found in Nursing Measures. Significant differences among professional roles were anticipated in this study, given the differing educational backgrounds and responsibilities in municipal healthcare [2], and the overall pattern is consistent with previous findings using the Ms. Olsen/NOP-CET instruments [12, 33]. However, in line with earlier research, the Nursing Measures domain showed smaller between-group differences. As reported by Bing-Jonsson et al. (2016) [12], this domain is influenced by the scoring key: ANs and assistants receive credit for selecting “consult an RN”, whereas RNs do not, which reduces separation between groups in this area.

This lack of significant differences between groups in nursing measures can be interpreted in the context of evolving service structures and reforms, where unclear task divisions in long-term care have blurred professional boundaries and complicated the definition, development, and assessment of competence across roles [5, 7]. This challenges the assumption that ANs consistently occupy a distinct intermediate position between RNs and assistants, indicating that holding a professional role does not itself guarantee the expected competence level. The findings underscore the importance of inclusive and accessible lifelong learning, as emphasized by United Nations Educational, Scientific and Cultural Organization (UNESCO) [1], to ensure that all healthcare personnel, regardless of role, have opportunities to improve the competence necessary for safe and effective care. In that regard, a structured CPD program like ClinObsMun highlights the potential for developing minimum competence among RNs, ANs and assistants through targeted and systematic training.

Across the three competence domains, the highest average number of correct answers was for the competence domain Acute Help with 3.7 out of 5 items, followed by Involving Physician with 3.5 out of 5 items, and Nursing Measures with 1.9 out of 7 items (Table 2). For the competence domain Acute Help, RNs had a mean of 4.4 correct answers out of 5 items. It can be debated whether it is appropriate for all professional groups, given the hierarchy of competence, to participate in the same educational content in the ClinObsMun program, as the lack of role-specific tailoring may reduce its effectiveness. The findings suggest that RNs are particularly well-prepared to handle acute situations, demonstrating the highest competence in acute care. Thus, the uniform training steps may be less relevant for some RNs, as they might perceive parts of the program as redundant due to their existing competence. Nevertheless, RNs have the potential to play a central role in supporting learning in the workplace by serving as role models and directly guiding and supporting ANs and assistants. Informal collegial consultation can provide peer feedback, foster the exchange of diverse perspectives, and play a vital role in sustaining and improving clinical performance for effective CPD in the workplace [6]. This aligns with Boud’s account of peer learning and reciprocal peer learning, in which participants learn with and from each other and provide peer feedback as part of the learning process [36]. Because clinical competence varies among individuals, workplace-based training is essential to ensure that all RNs, including those with less experience or outdated knowledge, stay aligned with current clinical practices. Tailored, locally anchored training can address these differences and support individual learning needs. Although shared courses may not fully accommodate the diverse levels of professional competence, they play a critical role in fostering a common language and mutual understanding among healthcare personnel, aligning with the Communities of Practice framework [19].

The domain Nursing Measures stands out because all three professional roles, RNs, ANs, and assistants, exhibited a low mean of correct answers. This domain involves recognizing and addressing subtle changes in a patient’s health status, which may not pose immediate acute threats but can indicate underlying chronic conditions or emerging health issues (Table 1). Homecare professionals found it challenging to use their observational competence to detect early signs of deterioration in frail older patients when symptoms were vague. Additionally, vital signs were measured infrequently, most often in relation to critical illnesses [37]. The findings highlight a need for workplace-based training that addresses both early detection and intervention, improving the observational competence of healthcare personnel in identifying deterioration in frail older patients. The ClinObsMun program is an example of a structured program for CPD that is designed to strengthen clinical observation skills and reinforce decision-making through systematic patient observation and the ABCDE methodology.

To summarize, the observed differences in competence among healthcare personnel underscore the importance of CPD, particularly in detecting and intervening in response to early signs of deterioration in frail older patients. As part of a lifelong learning approach, organizations should actively create supportive environments that foster continuous competence development [38, 39] and implement targeted initiatives to develop the competence of their health personnel [8, 40]. This requires anchoring not only at the organizational level but also through national policy frameworks, as underscored by UNESCO and WHO [1, 3, 4]. Training healthcare personnel in the use of NEWS2, ABCDE principles, and vital measurements included in the ClinObsMun program can strengthen their ability to detect early stages of deterioration in patients with preventable conditions. Such workplace-based training can ensure that healthcare personnel are better equipped to recognize and respond to subtle changes in patient health, thereby improving overall patient safety and care quality. Effective recognition of patient deterioration is a multifaceted process that requires an established workflow, accurate measurement of vital signs, the ability of healthcare professionals to interpret clinical data, and rapid-response services to ensure timely and appropriate treatment [37]. The ClinObsMun program supports this process through its comprehensive approach, which includes lectures, scenario-based training, and workshops designed to develop the skills necessary for effective detection and intervention in both acute and sub-acute settings. The program’s shared training steps are aligned with Experiential Learning Theory [17], engaging healthcare personnel in both hands-on learning and reflective practice. These shared experiences not only improve individual skills but also foster mutual understanding and collaboration across professional roles, which are essential for effective interdisciplinary teamwork. Thus, the ClinObsMun program represents a CPD initiative tailored to the actual learning needs of healthcare personnel.

### Contextual competence in care settings

To explore clinical competence at the contextual care‑setting level, this section compares home care and nursing homes. Regression analysis indicated higher competence among home‑care personnel than those in nursing homes. These findings suggest a shift in competence patterns that warrants investment in workplace‑based CPD across settings to ensure both home care and nursing homes are equipped to meet increasingly complex needs.

Contextual factors play a significant role in shaping clinical competence, particularly when comparing different care settings such as home care and nursing homes. Regression analysis indicated higher competence among home-care personnel compared with those in nursing homes for total competence and the domains Acute Help and Nursing Measures, with no workplace effect for Involving Physician. This finding is surprising, as it contrasts with earlier research reporting that nursing home personnel generally had higher levels of competence [12]. Considering the complexity of the patients’ health conditions and their typically short, expected lifespan in nursing homes [13, 35], it would be reasonable to assume that healthcare personnel’s competence, as well as the highest competence needs, would be found there. It is important to note, however, that the patient populations in home care and nursing homes are not fundamentally different, rather, they often represent different stages in the same illness trajectory. Therefore, when a patient receiving home care becomes older and more seriously ill, it is essential that they are not met by healthcare personnel with a lower competence level than those who previously provided their care at home.

Internationally, there has been a growing shift away from institutional care, with many countries focusing on expanding support services that allow especially older adults to receive care in their own homes [41]. Policies aimed at delivering care at the lowest effective level, such as favouring home-based services over institutional care, may unintentionally compromise the quality and viability of nursing homes. A comparative analysis of Ontario, Sweden and Norway discusses how such strategies can result in resource limitations and increased strain on long-term care facilities [7]. This highlights the importance of investing in competence development through workplace-based training across all care settings. As care provision continues to shift from institutions to home, a balanced approach is needed to ensure that both home care and nursing home services are equipped to meet the complex and evolving needs of an ageing population.

There is widespread agreement that in healthcare, continuous competence development is essential for patient safety and high-quality care [2–5]. Increasingly, the workplace is being recognised as a key arena for learning. Workplace-based training supports the ongoing development of clinical competence by embedding learning in everyday practice and enabling healthcare staff to respond dynamically to complex patient needs [8–10]. The ClinObsMun programme, developed for use in Norwegian municipal health services, aims to strengthen clinical competence in systematic patient observation and decision-making. In doing so, it responds to the WHO Global Patient Safety Action Plan 2021–2030, which highlights the importance of CPD to ensure that healthcare personnel are equipped to minimise risks to patient safety [3].

### Strengths and limitations

Nurse competence is often assessed through self-assessment. A strength of this study is that the Ms. Olsen instrument evaluates competence not through nurse self-satisfaction or self-criticism but based on the correctness of their action choices. Other strengths include the large sample size, a relatively high response rate, and the diversity of participants across variables such as workplace, professional group, position size, age, and work experience. In addition, the high share of respondents with more than five years of experience reflects the mature workforce in municipal long‑term care reported in Nordic settings [12, 35], thereby strengthening the study’s external validity by mirroring real‑world staffing profiles.

One limitation is that selecting only one answer from a set of multiple choices may be difficult, and it is unclear whether this accurately reflects on-the-job behaviour. However, this limitation is common to all tools designed to measure behaviour. Additionally, it is unknown who opted out of the competence measurement, meaning the potential bias created by non-response remains unclear. These findings may be generalisable to similar groups of municipal healthcare personnel in Norway, but caution is warranted in extending them to other professional groups or international settings.

The included predictors explained only 15% of the variation in total competence, with even lower explanatory power for specific domains: Acute Help at 14%, Nursing Measures at 6%, and Involving Physician at 4%. This suggests that other factors not included in our model may affect clinical competence, such as whether individuals work in supportive environments that promote learning cultures, where training and collaborative practices foster mutual understanding and strengthen support across professional roles. Both Experiential and Sociocultural Learning Theories emphasize that learning is strongly influenced by the context in which it occurs and the social environment surrounding the learner [17, 18], yet a supportive learning culture is challenging to measure through quantitative research. Therefore, qualitative research might provide deeper insights into how workplace culture influences competence development by capturing nuanced interactions and support dynamics that are difficult to quantify.

## Conclusion

This study revealed significant differences in clinical competence among healthcare personnel, with RNs consistently outperforming ANs and assistants across all domains. While RNs demonstrated strong acute care capabilities, the study also highlighted gaps in competence, particularly in Nursing Measures, where all professional roles exhibited low scores.

The regression analysis identified professional group, workplace, position size, and work experience as predictors of competence. However, these factors only explained a limited portion of the variation. This indicates that other unmeasured factors, such as the context of practice and organizational culture, may significantly impact competence levels.

To inform the development of workplace-based training initiatives, it is crucial to address these competence gaps through structured programs like ClinObsMun, which focus on systematic patient observation and improved decision-making. Tailoring training content to the specific needs of healthcare personnel and integrating reflective practices and peer collaboration will improve learning outcomes and ensure that all staff are equipped to meet complex patient needs effectively.

## Supplementary Information

Below is the link to the electronic supplementary material.

Supplementary Material 1: Appendix 1 includes the questionnaire items from the Ms. Olsen test and the corresponding answer key for registered nurses, assistant nurses, and assistants

## Data Availability

The datasets generated during the current study are not publicly available due to participant privacy concerns but are available from the corresponding author upon reasonable request.

## Abbreviations

CPD: Continuing Professional Development
RN: Registered Nurse
AN: Assistant Nurse
Ms. Olsen test: A questionnaire measuring clinical decision-making competence
NOP-CET: Nursing Older People Competence Evaluation Tool
STROBE: Strengthening the Reporting of Observational Studies in Epidemiology
NEWS2: National Early Warning Score 2
ABCDE: Airway, Breathing, Circulation, Disability, Exposure (clinical assessment method)
ClinObsMunClinical: Observation Competence in the Municipal Health Service program
WHO: World Health Organization
ICN: International Council of Nurses
UNESCO: United Nations Educational, Scientific and Cultural Organization
USHT: Centre for Development of Institutional and Home Care Services (Norway)
OECD: Organisation for Economic Co-operation and Development
ProACT: ProACT Norway – clinical assessment and response training
