# Supplementary material for: The importance of Continuing Professional Development of clinical competence in municipal healthcare: a cross-sectional study of workplace-based training
Source: BMC Nurs. 2026 Apr 15;25:499. doi: 10.1186/s12912-026-04546-7 (PMC13224509; doi:10.1186/s12912-026-04546-7)
Supplement: Supplementary file 1 — Supplementary Material 1: Appendix 1 includes the questionnaire items from the Ms. Olsen test and the corresponding answer key for registered nurses, assistant nurses, and assistants [file 12912_2026_4546_MOESM1_ESM.docx]

**Appendix 1**

| Item no | Item wording | Correct answer(s) for registered nurses | Correct answer(s) for assistant nurses and assistants |
| --- | --- | --- | --- |
| 1 | Patient has dyspnoea during rest within last two days | 5 | 4 or 5 |
| 2 | Patient coughs, has increased saliva, and respiration frequency above 20/min | 4 or 5 | 4 |
| 3 | Patient has irregular pulse increased more than 20/min in last two days | 4 | 4 |
| 4 | Patient has temperature above 38.5 | 4 or 5 | 4 |
| 5 | Patient is substantially dehydrated | 4 or 5 | 4 |
| 6 | Patient’s skin has rash, wounds, is red or itchy | 4 | 4 |
| 7 | Patient has reduced appetite and food intake | 3 or 4 | 3 or 4 |
| 8 | Patient is not able to eat | 4 or 5 | 4 |
| 9 | Patient has pain and discomfort in mouth | 4 | 4 |
| 10 | Patient is incontinent for urine, stings when urinates | 4 or 5 | 4 |
| 11 | Patient has much fresh blood in stool | 5 | 4 or 5 |
| 12 | Patient has increased needs to full care over last two days | 4 or 5 | 4 |
| 13 | Patient has fallen two times previous week | 4 or 5 | 4 |
| 14 | Patient has symptoms of partial paralysis | 6 | 4 or 6 |
| 15 | Patient is more tired during the day | 4 | 4 |
| 16 | Patient has changes in sight, hearing, speech, and comprehension | 6 | 4 or 6 |
| 17 | Patient has newly occurred chest pain | 6 | 4 or 6 |
| 18 | Patient has lost interest in keeping home in order, sleeps in chair instead of bed | 4 | 4 |
| 19 | Patient has short attention span and delusions | 5 | 4 or 5 |
| Response options: 1 = No action required, 2 = Observe again the following day, 3 = Consult a colleague, 4 = Nursing intervention immediately, 5 = Have the patient assessed by a physician today, 6 = Require acute help in hospital. | | | |
